# TRPV1 translocated to astrocytic membrane to promote migration and inflammatory infiltration thus promotes epilepsy after hypoxic ischemia in immature brain

**DOI:** 10.1186/s12974-019-1618-x

**Published:** 2019-11-13

**Authors:** Xin Wang, Xing-Liang Yang, Wei-Lin Kong, Meng-Liu Zeng, Lin Shao, Guang-Tong Jiang, Jing-Jing Cheng, Shuo Kong, Xiao-Hua He, Wan-Hong Liu, Tao-Xiang Chen, Bi-Wen Peng

**Affiliations:** 1 Department of Physiology, Hubei Provincial Key Laboratory of Developmentally Originated Disease, School of Basic Medical Sciences, Wuhan University, Wuhan, 430071 China; 2 Department of Pathophysiology, School of Basic Medical Sciences, Wuhan University, Wuhan, China; 3 Department of Immunology, School of Basic Medical Sciences, Wuhan University, Wuhan, China

**Keywords:** TRPV1, Inflammation, Astrocytes, HIBD, Epilepsy

## Abstract

**Background:**

Neonatal hypoxic-ischemic brain damage (HIBD), a leading cause of neonatal mortality, has intractable sequela such as epilepsy that seriously affected the life quality of HIBD survivors. We have previously shown that ion channel dysfunction in the central nervous system played an important role in the process of HIBD-induced epilepsy. Therefore, we continued to validate the underlying mechanisms of TRPV1 as a potential target for epilepsy.

**Methods:**

Neonatal hypoxic ischemia and oxygen-glucose deprivation (OGD) were used to simulate HIBD in vivo and in vitro. Primarily cultured astrocytes were used to assess the expression of TRPV1, glial fibrillary acidic protein (GFAP), cytoskeletal rearrangement, and inflammatory cytokines by using Western blot, q-PCR, and immunofluorescence. Furthermore, brain electrical activity in freely moving mice was recorded by electroencephalography (EEG). TRPV1 current and neuronal excitability were detected by whole-cell patch clamp.

**Results:**

Astrocytic TRPV1 translocated to the membrane after OGD. Mechanistically, astrocytic TRPV1 activation increased the inflow of Ca^2+^, which promoted G-actin polymerized to F-actin, thus promoted astrocyte migration after OGD. Moreover, astrocytic TRPV1 deficiency decreased the production and release of pro-inflammatory cytokines (TNF, IL-6, IL-1β, and iNOS) after OGD. It could also dramatically attenuate neuronal excitability after OGD and brain electrical activity in HIBD mice. Behavioral testing for seizures after HIBD revealed that TRPV1 knockout mice demonstrated prolonged onset latency, shortened duration, and decreased seizure severity when compared with wild-type mice.

**Conclusions:**

Collectively, TRPV1 promoted astrocyte migration thus helped the infiltration of pro-inflammatory cytokines (TNF, IL-1β, IL-6, and iNOS) from astrocytes into the vicinity of neurons to promote epilepsy. Our study provides a strong rationale for astrocytic TRPV1 to be a therapeutic target for anti-epileptogenesis after HIBD.

## Introduction

Neonatal hypoxia-ischemia brain damage (HIBD) remains a leading cause of severe neurological morbidity and mortality in neonates [1]. The mechanisms involved in brain damage include excitotoxicity, apoptosis, and glial overactivation [2]. Approximately 20% of newborns die in the postnatal period, 25% develop permanent neuropsychological sequelae such as epilepsy [3, 4], and 30% of children are unresponsive to conventional antiepileptic drugs due to resistance [5]. Thus, it is urgent to develop effective treatment method in HIBD-induced epilepsy.

TRPV1, a member of the vanilloid transient receptor potential (TRPV) channel family, was a Ca^2+^-permeable channel mostly studied as a pain receptor in sensory neurons [6, 7]. TRPV1 could be activated by a wide variety of exogenous and endogenous physical, chemical, and biological stimuli, such as hyperthermia (> 43 °C), low pH, capsaicin, endocannabinoid, and certain biotoxins [7, 8]. In the central nervous system, TRPV1 participates in synaptic transmission and neurogenesis [9]. However, its role in other cell types was poorly understood. Recent studies showed that TRPV1 was functionally expressed in glial cells, especially microglia and astrocytes [10, 11] and TRPV1 activation participated in neonatal brain injuries [12, 13].

Microglia TRPV1 activation upregulated the expression of pro-inflammation factors in febrile seizure mice [14]. As a member of innate immunity cells, astrocytes were also involved in the pathogenesis of neurological diseases and inflammatory responses [15–17]. TRPV1 deficiency exerted a neuroprotective effect on HIBD by mediating astrocyte activation and IL-1β release [18]. TRPV1 activation stimulated a JAK2-STAT3 pathway to regulate astrocytic activation, hypertrophy, and the expression of inflammatory cytokines (TNF, IL-1β, and IL-6) in vitro and in vivo [19–21]. Moreover, activation of neuroinflammatory signaling pathways in reactive astrocytes could promote the development of seizures [22, 23]. These results suggest that astrocyte-induced inflammatory response is a key factor in the pathogenesis of epilepsy. Besides, epileptic status attracted newly generated glia migrated to the regions of hippocampal damage [24], and attenuating the migration ability of astrocytes has a neuroprotective effect in ischemic brain injury diseases [25]. These indicate strongly that astrocyte migration ability might contribute to the further spread of proinflammatory factors to promote the incidence of epilepsy.

Taken together, TRPV1 was involved in the CNS inflammatory response [14] and epilepsy [26], which may lead to neurotoxicity [12]. Besides, the importance of microglial activation in hypoxia-induced neuroinflammation was well explored [27]. However, the detailed mechanism of astrocytic TRPV1 in epilepsy after HIBD has not been fully elucidated. Therefore, this study was aimed to investigate the role of TRPV1 on astrocytes in the epilepsy susceptibility after neonatal HIBD.

## Materials and methods

### Animal models and treatments

B6.129X1-TRPV1 KO mice used in the experiments were from the Jackson Laboratory [18]. Wild-type (WT) mice were provided by the Hubei Province Center for Animal Experiments. Mice were both on a C57BL/6J background. Animal experiments were approved by the Care and Use Committee of Wuhan University Medical School. All mice were housed at the animal facility of the Animal Biosafety Level III Laboratory (ABSL-III) of the Wuhan University with a suitable indoor environment. The day of the birth was defined as postnatal day zero (P0). Mice pups (*n* = 112) of P9 were used in this study and randomly divided into four groups (*n* = 28 each group): WT-Sham, WT-HI (hypoxic-ischemic), KO-Sham, and KO-HI.

### Neonatal hypoxia-ischemia model

Neonatal HI model was followed as previously described [28]. P9 mice were anesthetized, and their sterilized skin were incised; the right pulsating carotid artery was separated and cut off after its upper and lower ends had been tied. After 2 h of recovery, the pups were exposed in hypoxic environment (8% O_2_ in N_2_) at 37 °C for 45 min. Successful HI model showed significant edema in the ipsilateral hemisphere, while the sham group did not [18]. The mortality of this HI model was about 10%.

### Primary mouse cortical astrocytes and neuron cultures

Mouse cortical astrocytes and neuron cultures were prepared as previously described [29]. The cortex of P0 mice were isolated and digested in Hank’s balanced salt solution (HBSS) containing 0.05% trypsin at 37 °C for 6 min. Glial medium (1× Dulbecco’s modified Eagle’s medium (DMEM)/F12, 10% heat-inactivated fetal bovine serum, 1% L-glutamine, and 1% penicillin/streptomycin) were added to terminate the digestion. Dissociated cells were planted in T75 flasks with glial medium or in round glass with neuron medium (1× Neurobasal media, 2%B27, 1% L-glutamine) in cell culture incubator (37 °C, 5% CO_2_). Approximately 95% of the GFAP-positive or 95% of the neuron-positive cells were used for the experiment (data not shown).

### OGD progression

Oxygen-glucose deprivation (OGD) was conducted as described previously [30]. Astrocytes were grown in glial media for 10 days, and all groups of glial medium were changed to serum-free glial medium 24 h before OGD. The media of OGD groups were changed to OGD medium (serum- and glucose-free DMEM). The plates were placed in a hypoxic/anoxic chamber with 1% O_2_, 5% CO_2_, and 94% N_2_ for 3.5 h at 37 °C, and then were removed from the anaerobic chamber and the OGD medium was changed to fresh glial media. The media of control groups also changed to fresh glial media remained in a regular incubator.

### Quantitative real-time PCR

Total RNA was extracted from the cortical-derived astrocytes to detect the mRNA level of TNF (tumor necrosis factor), IL-1β (interleukin 1β), IL-6 (interleukin 6), inducible nitric oxide synthase (iNOS), arginase-1 (ARG-1), H2-T23, Iigp1, Fkbp5, S100a10, Cd109, and Emp1 (Sangon Biotech, Shanghai, Co., Ltd.). Real-time PCR was performed on the SYBR-Green premix based on the manufacturer’s specification. The cycling parameters for the CFX96 sequence detection system (CFX Connect of Wuhan University Institutional Center) were 95 °C for 5 min, 35 cycles of 95 °C for 10 s, 58 °C for 20 s, and 72 °C for 20 s. The expression of target genes was normalized to the mRNA level of β-actin as an internal control. The ΔΔCt values of each group were analyzed, and the mRNA expression of different groups was normalized to 2^−ΔΔCt^. The primers used in this experiment are listed in Table 1 (Additional file 1).

**Table 1:** q-PCR primer sequences applied

Primer	Forward primer 5′-3′	Reverse primer 5′-3
β-actin	CACGATGGAGGGGCCGGACTCATC	TAAAGACCTCTATGCCAACACAGT
TRPV1	CGAGGATGGGAAGAATAACTCACTG	GGATGATGAAGACAGCCTTGAAGTC
IL-1β	GCAGTGGTTCGAGGCCTAAT	CTCATCACTGTCAAAAGGTGGC
IL-6	ATTTCCTCTGGTCTTCTGGAGT	TCTGTGACTCCAGCTTATCTCTTG
TNF	GAGGCACTCCCCCAAAAGAT	GGCCATTTGGGAACTTCTCATC
iNOS	TGGTGAAGGGACTGAGCTGT	CTGAGAACAGCACAAGGGGT
Arginase-1	CAACGGGAGGGTAACCATAAG	GAAAGGAACTGCTGGGATACA
H2-T23	GGACCGCGAATGACATAGC	GCACCTCAGGGTGACTTCAT
Iigp1	GGGGCAATAGCTCATTGGTA	ACCTCGAAGACATCCCCTTT
Fkbp5	TATGCTTATGGCTCGGCTGG	CAGCCTTCCAGGTGGACTTT
S100a10	GAAAGGGAGTTCCCTGGGTT	CCCACTTTTCCATCTCGGCA
Cd109	GTCGCTCACAGGTACCTCAA	CTGTGAAGTTGAGCGTTGGC
Emp1	ACCATTGCCAACGTCTGGAT	TGGAACACGAAGACCACGAG

### Western blot

Astrocytic proteins were extracted, and the protein concentrations were measured. Equal amounts of protein were loaded on an SDS-PAGE gel. After electrophoresis and transfer to a polyvinylidene fluoride (PVDF) membrane, the membranes were blocked by 5% skimmed milk for 2 h. The membranes were incubated with the anti-TRPV1 (Novus biologicals, #9886, 1:1000), anti-GFAP (Cell Signaling, #3670, 1:1000), anti-GAPDH (Abcam, #9485, 1:1000), anti-ARG-1(Cell Signaling, #9819, 1:1000), and anti-β-actin (Protein tech, #60008, 1:10000) antibody overnight at 4 °C. Corresponding secondary antibodies were incubated for 1 h at room temperature. The reaction was detected in a chemiluminescent reagent (ECL; Menlo Park, California, USA).

### Measurement of cytokine by enzyme-linked immunosorbent assay

The protein concentrations of IL-1β (purchased from 4A Biotech Co., Ltd), IL-6 (purchased from Bioswamp, MU30044), and TNF (purchased from Bioswamp, MU30030) in the supernatants of astrocytes were quantified using enzyme-linked immunosorbent assay (ELISA) kits according to the manufacturer’s instruction. The supernatant was added into a 96-well plate and incubated with the corresponding primary antibody (90 min, 37 °C). After washing three times with wash buffer, the secondary antibody was used to incubate for 60 min at 37 °C. Finally, the absorbance at 450 nm was recorded.

### Scratch-wound assay

Scratch-wound assay was conducted as described previously [31]. A single scratch with a 1-mL sterile pipette tip was made through the mostly confluent astrocyte monolayer, and cells were washed and maintained in serum-free glial media. Images were acquired at 24 h after scratching using a fluorescence microscope (Olympus IX 73 DP80).

### Cell viability assay

Cell counting kit-8 (CCK-8) assay was performed as previously described [18]. Astrocytes were seeded at a density of 8 × 10^3^ mL^−1^. Cell viability was subsequently assessed using the CCK-8 (Dojindo, Japan). The optical absorbance at 450 nm was detected.

### Immunofluorescence

Immunofluorescence staining was carried out to detect GFAP expression in mice after HIBD as well as TRPV1, GFAP, and iNOS expression in astrocytes after OGD. Brain serial coronal sections and astrocytes were washed with PBS before fixed with 4% paraformaldehyde at room temperature for 30 min. Subsequently, they were incubated with a blocking solution (5% FBS) for 30 min at 37 °C. Then, they were incubated with the anti-TRPV1 (Novus Biologicals, #9886, 1:300), anti-GFAP (Cell Signaling, #3670, 1:200), or anti-iNOS (Cell Signaling, #2985, 1:200) antibodies overnight at 4 °C. On the following day, they were washed and incubated with secondary antibodies Cy3-conjugated anti-IgG (Protein tech, SA00009-4, 1:30), Alexa Fluor® 488 Conjugates (Cell Signaling Technology, #4408, 1:200) for 1 h at 37 °C and DAPI (Beyotime, #C1002,1:2000) for 1 min at room temperature. Phalloidin staining was performed following the same protocol used for immunofluorescence (IF) while astrocytes were incubated with FITC-phalloidin (sigma, #p5282, 5 μg/ml) for 1 h and DAPI for 1 min at 37 °C in the dark. Images were obtained using a confocal microscope (Leica-LCS-SP8-STED).

### Measurement of intracellular Ca^2+^ concentration

Intracellular Ca^2+^ concentration [Ca^2+^]*i* was measured by using the Ca^2+^ binding dyes Fluo-3 AM (BBcellProbe, BB-48112, 1:1000 diluted with HBSS). Cells were incubated with Fluo-3 AM for 30 min at 37 °C in dark. Then, cells were washed with PBS and incubated for a further 30 min in HBSS at 37 °C. Images were obtained by fluorescent microscope (Leica).

### F-actin to G-actin ratio

To analyze the cytoskeletal rearrangement of astrocytes, the F-actin to G-actin ratio was determined as previously described [32]. The two forms of actins differ in that F-actin is insoluble while G-actin is soluble. Astrocytes were homogenized in cold lysis buffer and centrifuged (15,000*g*, 20 min). Soluble actin (G-actin) was measured in the supernatant. The insoluble F-actin in the pellet was resuspended in lysis buffer plus an equal volume of buffer 2 and incubated on ice for1 h to convert F-actin into soluble G-actin. The samples were then centrifuged again to get F-actin. Samples from G-actin and F-actin fractions were proportionally loaded and analyzed by Western blotting using a specific actin antibody (Millipore, #MAB1501, 1:10000).

### Morphological assessment

Images (Olympus U-HGLGPS) were imported into ImageJ for morphological analysis. Detailed information about the branch length of each cell could be analyzed using a plugin named Analyze Skeleton [33]. Cell soma size was measured by selection outlining of each cell soma and selecting the “area” analyses to measure.

### Electrode implantation and electroencephalograph recording

Electroencephalograph (EEG) was conducted to record the role of TRPV1 in electrical activity of the brain in freely moving mice. The mice were anesthetized and then fixed into the stereotaxic apparatus. The bipolar twisted silver steel electrodes were embedded in the skull with dental cement. These electrodes were implanted into the bilateral hippocampal CA3 (2.3 mm posterior to bregma, 2.1 mm lateral to sagittal suture, 2 mm ventral to the dura mater). The spontaneous EEG seizures were induced by pentylenetetrazole (PTZ, 30 mg/kg) and examined for 30 min. The EEG signals were digitized with Lab Chart software (AD Instruments). Seizure severity was classified into five levels [34]: (I) facial movement, (II) head nodding, (III) unilateral forelimb clonus, (IV) bilateral forelimb clonus, and (V) tonic-clonic seizure, rearing, failing, or death.

### Patch clamp recordings

Whole-cell current recordings (Wuhan University Institutional Center) were used to detect the TRPV1 current of astrocytes and the electrical activity of neuron with a AXON 700B and Digidata 1550 (Axon, Molecular Device) at 22 ± 2 °C. Bath solution (in mM) consists of 140 NaCl, 5 KCl, 2 MgCl_2_, 2 CaCl_2_, 10 HEPES, and 10 glucose (pH 7.4 adjusted with NaOH). The pipette (4~6 MΩ) was filled with the intracellular solution (in mM): 140 CsCl, 5 EGTA, and 10 HEPES (pH 7.2 adjusted with CsOH). AP (action potential) was evoked by a series of current pulses from − 50 to + 100 pA with a step size of 15 pA. For recording capsaicin (CAP)-induced current in astrocytes, drugs were delivered using a gravitational perfusion system (ALA-VM8, Scientific Instruments) with 3–4 mL/min. CaCl_2_ was removed from the bath solution to reduce the desensitization of TRPV1 channel caused by CAP [35]. Series resistances (Rs) were monitored throughout the experiment and normally < 20 MΩ. Electrophysiological data were filtered at 1.0 kHz and digitized at 50 kHz. Dates were collected with pClamp 10.4 software (Axon Instruments).

### Experimental design and statistical analysis

GraphPad Prism 7 (RRID: SCR_002798) software was used to analyze dates and form graphs in this work (including which tests were performed, exact *P* values, and sample sizes). Statistical differences between groups were analyzed with either an unpaired *t* test or one-way analysis of variance (ANOVA) where appropriate. At least three independent experiments were applied to collect effective data. Bias was avoided by making sure that the assessor was blinded to collecting and analyzing data. *P* < 0.05 was considered significant. All values are presented as the mean ± standard error of the mean (SEM).

## Results

### TRPV1 promoted epilepsy following neonatal HIBD

**Fig. 1:**
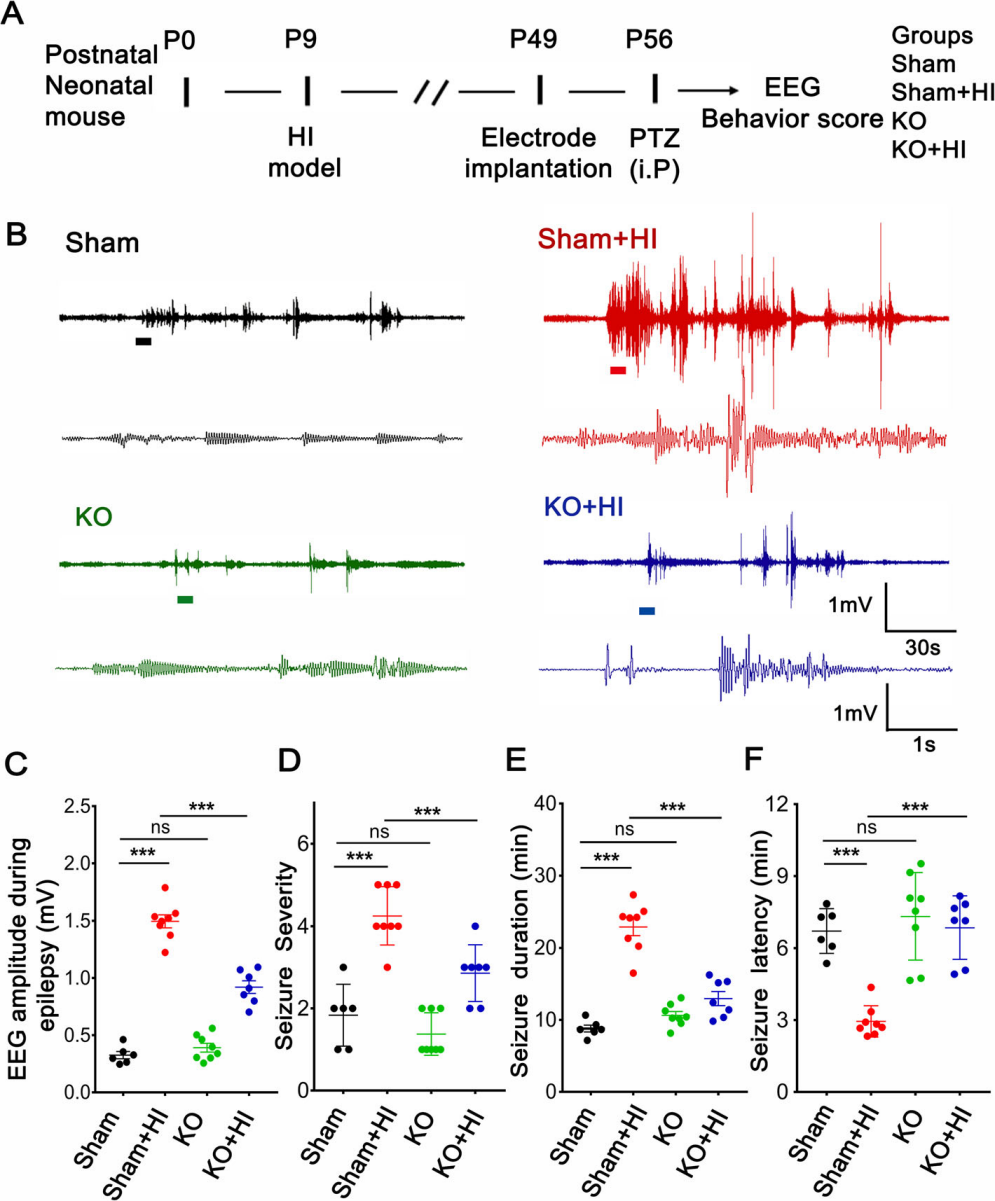
TRPV1 knockdown decreased spontaneous EEG seizures following neonatal HIBD. **a** Diagram of the experimental design. **b** Representative traces of EEG recordings and the corresponding enlarged part from the sham (black trace), HI (red trace), KO (green trace), and KO+OGD (blue trace). **c** The amplitude of the EEG recording after PTZ treatment. **d** The intensity stage of seizure. **e** The duration of seizure. **f** The tendency of seizure latency. *n* = 6~8 mice per experimental group. ns mean no significance. ***P < 0.001. Tukey’s test after one-way ANOVA

To determine the effects of TRPV1 on PTZ-induced seizure susceptibility after neonatal HIBD, neonatal mouse model of HIBD was established (Fig. 1a) and spontaneous EEG recording (Fig. 1b) and behavioral observation were used to assess the epilepsy susceptibility. The amplitude of spikes in the mice after HIBD was far greater than that in the sham group of mice, and it showed no obvious difference between the sham group and KO group, whereas behavioral testing showed TRPV1 deficiency in mice which markedly reduced the amplitude (Fig. 1c), the severity (Fig. 1d), and the duration (Fig. 1e) but increased the latency (Fig. 1f) of seizure compared with WT mice after HIBD. Taken together, these findings indicate that TRPV1 knockout was able to decrease PTZ-induced seizure after mice neonatal HIBD.

### TRPV1 translocated to the member in astrocytes after OGD

**Fig. 2:**
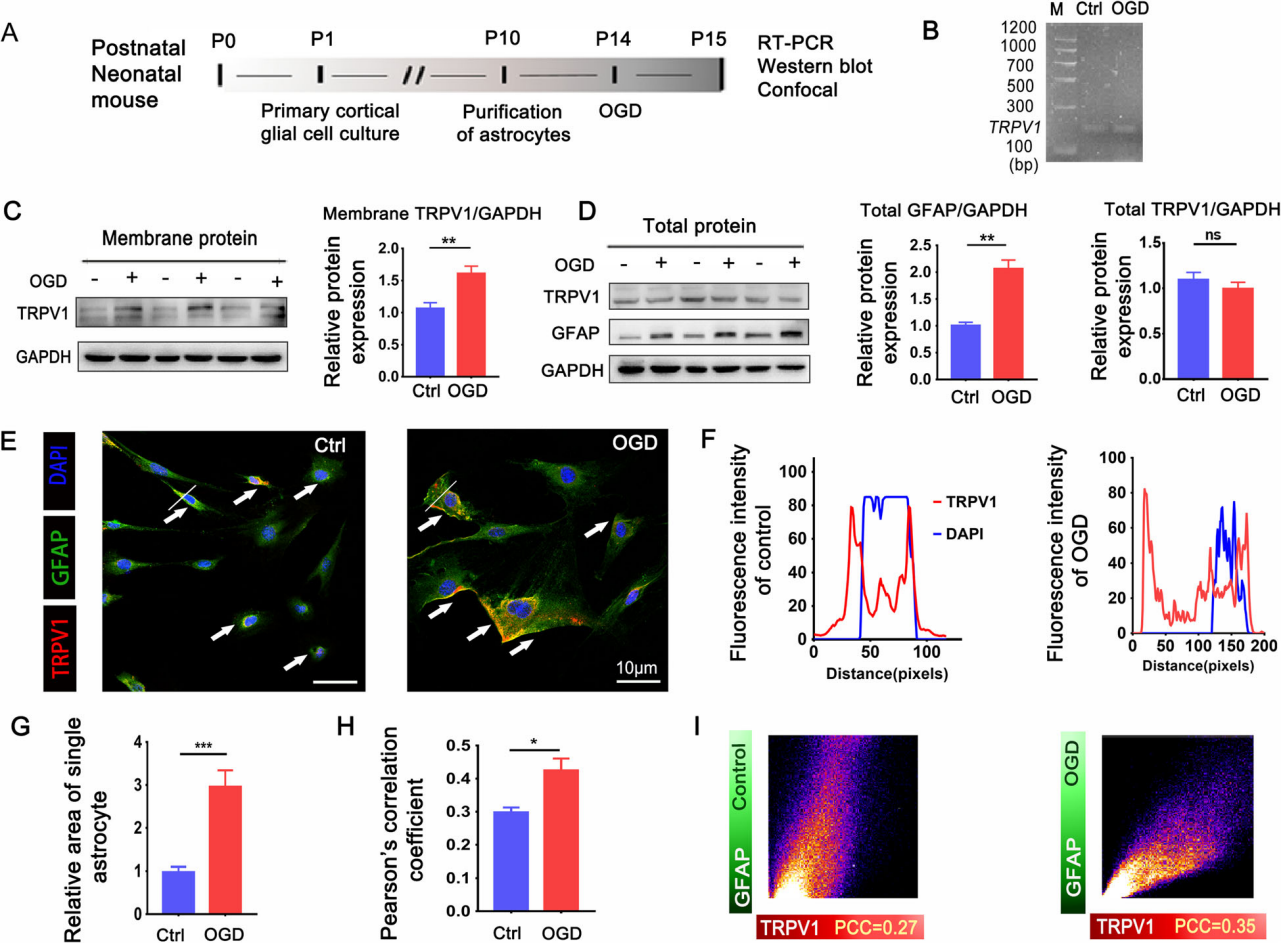
Expression and distribution of TRPV1 in primary cultured astrocytes after OGD. **a** Schematic of experimental design. **b** TRPV1 transcripts were detected in astrocytes by RT-PCR. M: DNA marker. **c** The membrane protein of TRPV1 in immunoreactive bands and quantitative analysis after OGD. **d** Western blots and quantification for GFAP and TRPV1 of total protein in astrocytes. **e** Confocal images showed cellular localization patterns of TRPV1 (red) in GFAP (green) astrocytes with the nucleus stained with DAPI (blue). White arrows indicated the distribution of TRPV1 in astrocytes. White line analyzed the distance between TRPV1 and the DAPI in astrocytes and analyzed in **f**. The intensity of GFAP was determined in 5 fields/well and divided by the number of cells counterstained with DAPI. **g** Relative size of astrocyte. **h** Co-localization between GFAP and TRPV1 by using the PCC method. **i** The intensity and distribution of different colored pixels of GFAP (green) and TRPV1 (red) in astrocytes. *P < 0.05, ** *P* < 0.01 ***P < 0.001. Scale 10 μm. All data: mean ± SEM. Unpaired Student’s *t* test

To explore whether TRPV1 was involved in spontaneous seizures mediated by reactive astrocytes, the expression of TRPV1 in astrocytes after HIBD was firstly explored. RT-PCR examined TRPV1 existed in astrocytes (Fig. 2b), and Western blot showed no significant change in the total protein expression of TRPV1 after OGD (Fig. 2d). Intriguingly, the expression of TRPV1 on the membrane of cultured astrocytes was upregulated after OGD (Fig. 2c). Confocal fluorescence further confirmed that TRPV1 more distributed on the astrocytic plasma membrane after OGD (Fig. 2e, f). The size of astrocyte increased after OGD (Fig. 2g), combined with the increased expression of GFAP in Fig. 2d; it suggests that astrocyte transformed into reactive astrocytes. Representative co-localization of double-labeled staining in GFAP and TRPV1 was quantified by Pearson correlation coefficient (PCC), and PCC was increased in astrocytes which explained the correlation between TRPV1 and GFAP was enhancement after OGD (Fig. 2h, i). Collectively, these results suggest that TRPV1 channels are expressed in mice cortical astrocytes and translocated to the membrane of reactive astrocytes after OGD.

### TRPV1 activation increased intracellular Ca^2+^, which promoted G-actin polymerized to F-actin, to promote astrocyte migration after OGD

**Fig. 3:**
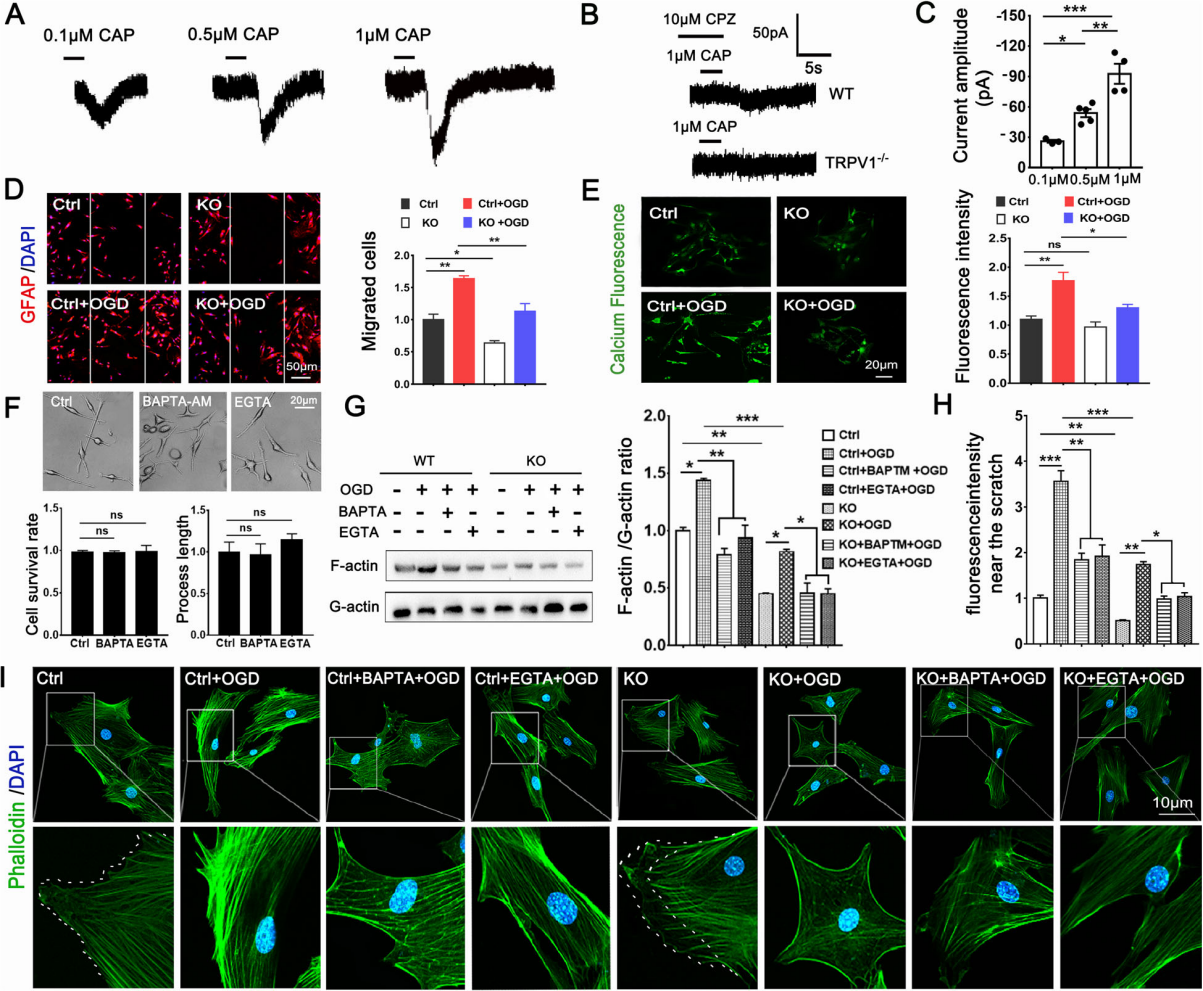
TRPV1 promoted astrocyte migration after OGD. **a** Whole-cell patch clamp detected TRPV1-like currents induced by 0.1 μM, 0.5 μM, and 1 μM CAP in WT astrocytes. **b** WT astrocytes which pre-administered with 10 μM CPZ and TRPV1 knockout astrocytes were treated with 1 μM CAP separately to detected TRPV1 current. **c** TRPV1 current induced by different concentrations of CAP. Fluorescence images and bar chart showed astrocytes post-scratch (**d**) and loaded with Fura-3 AM (**e**). **f** Differential interference contrast images were taken to record the morphology of astrocytes. Bar chart showed cell survival rate and cell process length. **g** Western blotting and histogram revealed the ratio of F-actin to G-actin. Confocal images (**i**) and bar chart (**h**) showed fluorescence intensity at the leading edge of astrocytes near the scratch area. High magnification views of boxed areas are shown in the bottom. Scale 50 μm for (**d**), 20 μm for (**e**), and 10 μm for (**i**). Average values represent the mean ± SEM. *P < 0.05, ** *P* < 0.01, ***P < 0.001 (Tukey’s test after one-way ANOVA)

To investigate the role of TRPV1 on astrocyte migration, we first performed whole-cell patch clamps to investigate whether TRPV1 acted as a capsaicin-sensitive ion channel on astrocytes. We demonstrated TRPV1 current was present on astrocytes with concentration-dependent capsaicin (Fig. 3a–c). Scratch experiments showed TRPV1 deficiency attenuated astrocyte migration ability (Fig. 3d). We next used Ca^2+^ binding dyes Fluo-3/AM to analyze astrocytic [Ca^2+^]*i* and found TRPV1 knockout decreased [Ca^2+^]*i* after OGD (Fig. 3e). Besides, neither EGTA nor BAPTA affected the morphology, the survival rate, and the process length of astrocytes (Fig. 3f). We then probed the molecular mechanism of TRPV1 in actin dynamics of astrocytes. The ratio of F-actin to G-actin, reflecting the balance between actin polymerization and de-polymerization, was significantly decreased in TRPV1^−/−^ astrocytes compared with the control astrocytes (Fig. 3g).

To further confirm changes in the subcellular localization of cytoskeletal proteins during migration, the localization of F-actin cytoskeleton in astrocytes near the scratch area was examined. Astrocytes showed clearly defined microtubules, and the localization of microtubules was most intense and directly migrated toward the leading edge after OGD. Weakened microtubule migration was found when treated with EGTA or BAPTA. Interestingly, the microtubule of TRPV1^−/−^ astrocytes failed to extend to the periphery of the cell (Fig. 3i, h). These data indicate that TRPV1 promoted the migration of astrocytes by regulating Ca^2+^ inflow to mediate the skeleton protein G-actin polymerized into F-actin after OGD.

### TRPV1 deficiency blocked the production and release of inflammatory factors in astrocytes after OGD

**Fig. 4:**
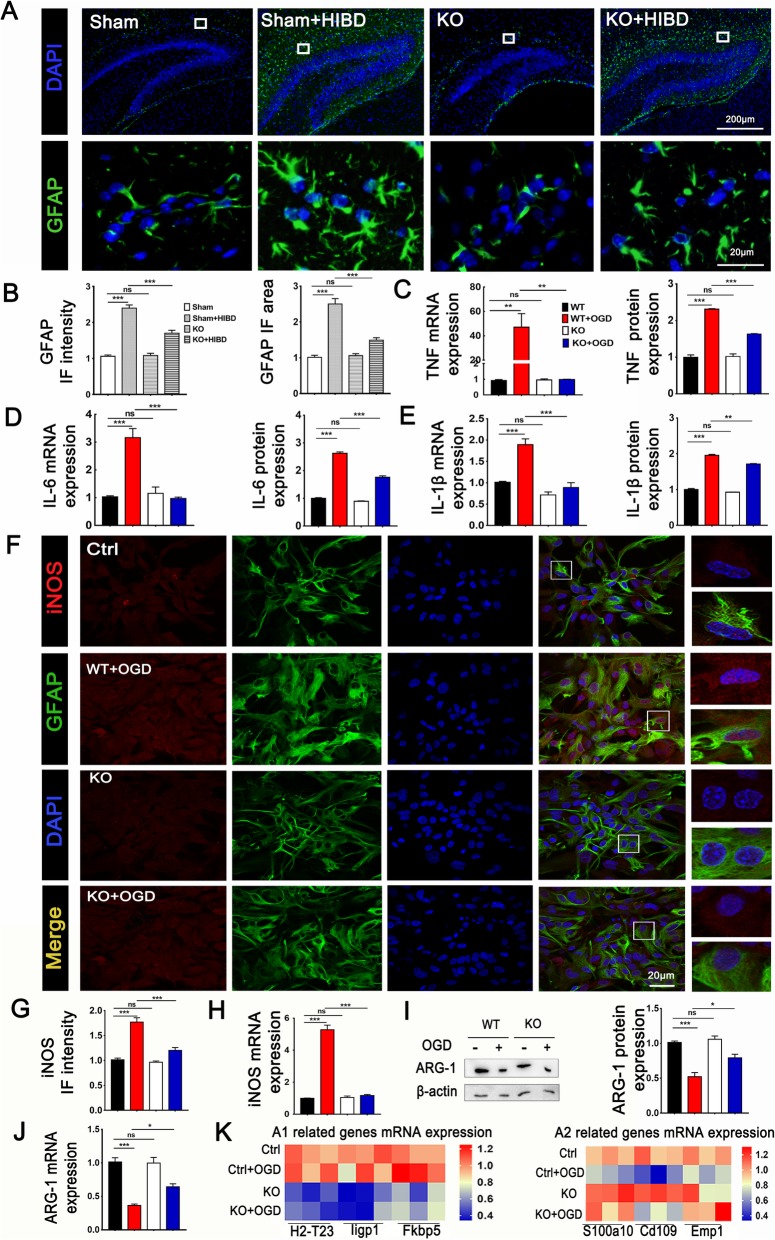
TRPV1 promoted the production and release of inflammatory factors in astrocytes after OGD. **a** IF (scale 200 μm) and white squares corresponding enlarged view (scale 20 μm) showed the IF intensity and area of GFAP (**b**). Relative mRNA levels and the protein expression of TNF (**c**), IL-6 (**d**), and IL-1β (**e**). **f** IF staining of astrocytes (scale 20 μm) for iNOS (red), GFAP (green), and DAPI (blue). Co-localization areas were marked by white squares and were enlarged below the right side; co-location between iNOS and DAPI was above it. Histogram analyzed the iNOS relative IF intensity (**g**) and mRNA expression (**h**). **i** Western blot and quantification showed the protein expression of ARG-1. **j** q-PCR analyzed the mRNA expression of ARG-1. **k** q- PCR detected the expression of A1-related genes (H2-T23, Iigp1, Fkbp5) and A2-related genes (S100a10, Cd109, Emp1). Data are shown as the mean ± SEM; *P < 0.05, **P < 0.01, ***P < 0.001, *n* = 3 per group, based on a one-way ANOVA

To analyze the role of TRPV1 on inflammatory response of astrocytes, the changes in the mRNA and protein levels of inflammatory cytokine of astrocytes were evaluated. TRPV1^−/−^ significantly reduced the IF intensity and IF area of GFAP in the brain tissue after HIBD (Fig. 4a, b). Besides, TRPV1 deficiency clearly reduced the level of mRNA and protein of TNF (Fig. 4c), IL-6 (Fig. 4d), and IL-β (Fig. 4e) at 24 h compared with control astrocytes after OGD. In addition, we also detected the expression of ARG-1 and iNOS which corresponds to the astrocytic anti-inflammatory and pro-inflammatory status [36]. TRPV1 genetic ablation reversed the increased intensity (Fig. 4g) and mRNA expression (Fig. 4h) of iNOS after OGD. Correspondingly, the protein (Fig. 4i) and mRNA expression (Fig. 4j) of ARG-1 were increased in TRPV1^−/−^ astrocytes after OGD. These results indicate that astrocytic TRPV1 knockout reduced the production and release of pro-inflammatory factors. To explore the role of TRPV1 on neurotoxicity A1-like astrocytes and neuroprotective A2-like astrocytes, the specifically related genes of both phenotypes were randomly selected. TRPV1 deficiency downregulated A1 astrocyte-related genes and upregulated A2 astrocyte-related gene expression compared with control astrocytes after OGD at 24 h (Fig. 4k). Taken together, these results demonstrated TRPV1 deficiency blocked pro-inflammatory cytokine production and release, and TRPV1 was involved in modifying A1/A2-related gene expression in astrocytes following OGD.

### Astrocytic TRPV1 promoted neuronal excitability

**Fig. 5:**
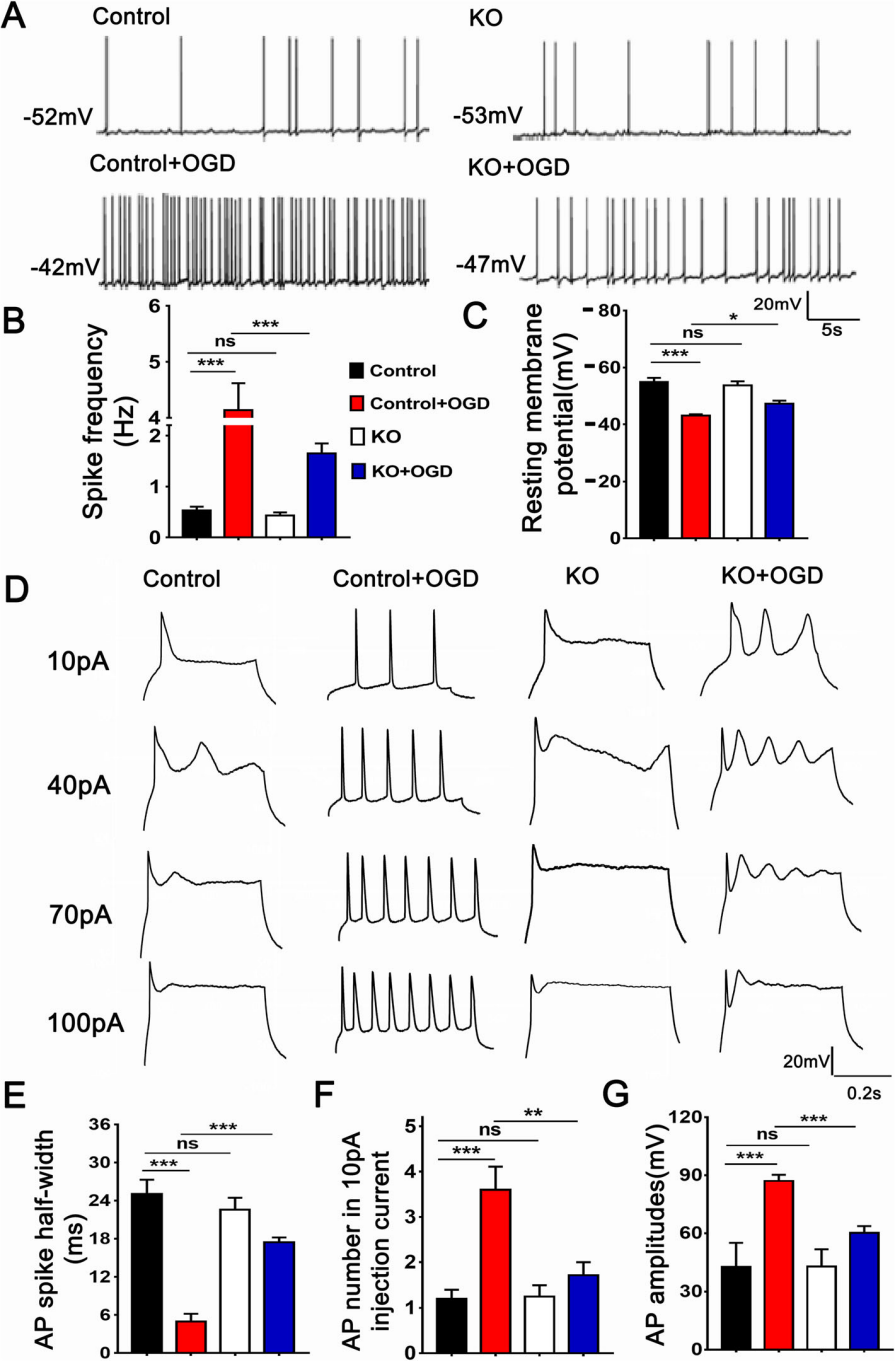
Astrocytic TRPV1 promoted neuronal excitement after OGD. Whole-cell patch clamps were used to detect spontaneously AP (**a**), induced AP (**b**), and RMP (**c**) of the neurons pre-treated with the astrocytes medium supernatant. Histogram showed spike frequency (**d**), the spike half-width of AP (**e**), the number of AP firing in 10 pA injection current (**f**), and the amplitudes of AP (**g**) of each groups. *n* = 3~5 per group. *P < 0.05,  **P < 0.01, ***P < 0.001, *n* = 3 per group, based on a Tukey’s test after one-way ANOVA. Data are shown as the mean ± SEM

To detect the effect of astrocytic TRPV1 on neuronal excitability in vitro, the medium supernatant of astrocytes in control, control+OGD, KO, and KO+OGD was obtained to pre-incubate neuron for half an hour. Voltage patch clamp was used to detect the RMP (resting membrane potential), spontaneous AP, and induced AP on neurons. TRPV1 deficiency remarkably decreased the frequency of spontaneous AP (Fig. 5a, b) and the degree of neuron depolarization compared with that of control astrocytes after OGD (Fig. 5c). Moreover, current patch clamp was performed to record the induced AP by injecting a series of depolarizing current (10 pA, 40 pA, 70 pA, 100 pA). Ten-picoampere current injection-induced AP was analyzed. Consistent with the results of voltage clamp, TRPV1 deficiency not only successfully increased the half-wave width (Fig. 5e), but lessened the firing number (Fig. 5f) and the amplitude of AP (Fig. 5g) with that of control astrocytes after OGD. Thus, astrocyte with TRPV1 deficiency lost the ability to promote neuronal discharge and excitation after OGD.

## Discussion

Epilepsy is one of the most common sequelae of neonatal HIBD [37]. We have previously confirmed TRPV1 deficiency played a neuroprotective role on neonatal HIBD [18]. However, the role of TRPV1 in epilepsy susceptibility after neonatal HIBD is unknown. In the present study, we demonstrated that TRPV1 deficiency reversed OGD-induced increase in neuronal excitability comparing with control astrocytes and HIBD-induced increased epilepsy susceptibility comparing with WT mice. Specifically, TRPV1 translocated to the astrocytic membrane after HIBD, promoting astrocyte migration and inflammatory infiltration, thereby increasing neuronal excitability and promoting the onset of epilepsy.

Since the last decade, epilepsy studies have focused on changes in neuronal activity and excitability; such neuron-centered study ignores the role of glial cells and involvement of neuroinflammation in the pathogenesis of epilepsy [38–40]. Our previous research showed microglia TRPV1 played important role in febrile seizures [14], while the contribution of astrocytic TRPV1 channel in epileptic susceptibility after HIBD is not well defined. In this study, we identified that the TRPV1 channel is functionally expressed in astrocytes and TRPV1 on astrocytes promoted neuronal excitability after OGD. By employing the whole-cell patch clamp technique, we verified the functionality of the TRPV1 channel at the astrocytic plasma membrane. However, the TRPV1 current in astrocytes was much smaller compared to that previously reported in sensory neurons [41, 42], a comparatively lower TRPV1 expression level in astrocytes might account for it. Nonetheless, small currents could also produce physiologically significant increases in [Ca^2+^]*i* and have profound consequences on cell physiology [43]. Accordingly, [Ca^2+^]*i* was detected and finally revealed TRPV1 promoted the astrocyte migration by regulating Ca^2+^ inflow.

The phenomenon that TRPV1 contributed to the astrocyte migration we found in this study was similar with those of Karen W. Ho [31]. Comparatively, with the administration of TRPV1 inhibitor CPZ to inhibit the function of TRPV1 in WT astrocytes, the TRPV1^−/−^ astrocytes we used could totally avoid the side effects from the drug. In terms of the molecular mechanism of migration, we investigated TRPV1 deficiency reducing Ca^2+^ inflow thus decreasing G-actin polymerized into F-actin. Finally, a scratch assay was performed based on the OGD model to investigate the effect of TRPV1 on the astrocyte migration.

Directed cell migration requires local Ca^2+^ pulses near the leading edge of the cell to activate myosin and to modulate focal adhesions [44–46]. We showed that OGD-treated TRPV1^−/−^ astrocytes significantly upregulated [Ca^2+^]*i* and skeletal protein polymerization compared with the untreated TRPV1^−/−^ astrocytes. Besides TRPV1, other proteins might also existed in astrocytes and played the similar role. Consistent with our speculation, TRPV4 channels, as well within TRP family, were also functionally expressed in astrocytes and regulating Ca^2+^ influx [47]. L-type voltage-regulated calcium channels (VOCCs) Cav1.2 could also mediate astrocyte Ca^2+^ influx, inhibited astrocyte activation and migration [48]. Therefore, whether TRPV4 and Cav1.2 channel were activated in neonatal HIBD thus promoting astrocyte migration is also worthwhile further confirmation.

TRPV1 has a role in promoting astrocyte migration thereby facilitating the spread of inflammation. Neuroinflammation has been reported to be deeply involved in the pathological process of HIBD deterioration [49, 50]. Besides, neuroinflammation has been detected not only in animal models of seizures, but also in children with drug-resistant epilepsy [51]. TRPV1 played an important role in neuroinflammation-induced seizures; it not only was a novel detector and biomarker of brain inflammation, but also regulated microglia-neuron communication by promoting neuroinflammation and disrupting brain homeostasis [10, 52, 53]. Therefore, we focus on the role of astrocytic TRPV1 in neuroinflammation in HIBD-induced epilepsy.

Consistent with previous results, we found that astrocytic TRPV1 increased the production of pro-inflammatory cytokines IL-1β after OGD (24 h) and promoted astrocyte activation after HIBD (24 h) [18]. Moreover, we demonstrated TRPV1 played a pro-epileptogenesis role in HI mice model (24 h) due to the result that epilepsy susceptibility was significantly decreased in TRPV1 deficiency mice compared with WT mice after HIBD. Furthermore, astrocytic TRPV1 exerted neurotoxicity role in releasing excessive pro-inflammatory factors such as IL-6, IL-1β, TNF, and iNOS after OGD. Hence, TRPV1, a crucial pivot for astrocytes involved in the inflammatory response, promoted astrocytic pro-inflammatory state and aggravated the brain inflammatory microenvironment to promote the onset of seizures after HIBD. While which inflammatory factor plays the most significant role or whether multiple inflammatory factors have synergistic effects in the pathogenesis of epilepsy needs further exploration.

Microglial activation is often categorized as either M1 phenotype (referred to as pro-inflammatory) or M2 phenotype (referred to as anti-inflammatory) [54–56]. There is considerable evidence indicating that microglial M1 markers are overexpressed in epileptic foci and cerebrospinal fluid (CSF) [57]. Activation of microglial TRPV1 promoted the release of excitotoxicity pro-inflammatory factors, which was a critical etiology of seizures [52, 58]. Moreover, activated microglia could also release anti-inflammatory factors like TGF-β1 (transforming growth factor-beta1), which could convert the polarization states of microglia from M1 phenotype to M2 phenotype [54, 55, 59]. However, it is unknown whether TRPV1 regulated astrocytic phenotype-related gene expression changes in HIBD-induced seizures. Reactive astrocytes are classified into A1 phenotype (referred to as neurotoxicity) or A2 phenotype (referred to as neuroprotective) [60]. In this study, we demonstrated that TRPV1 deficiency downregulated A1 astrocyte-related genes and upregulated A2 astrocyte-related gene expression compared with control astrocytes after OGD at 24 h (Fig. 4k). It demonstrated TRPV1 was involved in modifying A1/A2-related gene expression in astrocytes following OGD. However, whether TRPV1 regulated astrocytic phenotype changes such as A1 phenotype changed to A2 phenotype in HIBD-induced seizures deserves further study.

**Fig. 6:**
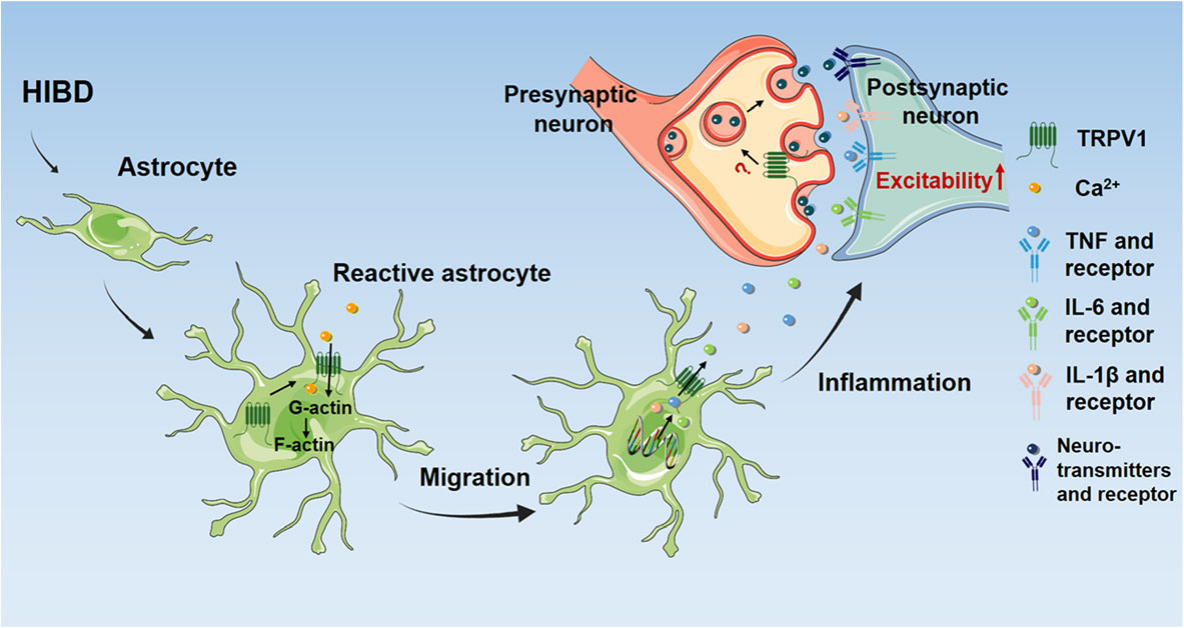
Schematic diagram depicting the proposed mechanism involved in HI-induced. The evolution of seizures after HI progresses along the following steps: TRPV1 activated and transported to the astrocytic plasma membrane after HIBD, and it regulated Ca2+ inflow, mediating G-actin polymerized to F-actin, promoting astrocyte migration thus contribute to further spread of proinflammatory factors into the vicinity of neurons to promote the development of seizure. A red question mark indicates problems needed to be solved, and the detailed contents were explained in the discussion section

The appropriate subcellular localization of proteins provides the physiological context for their function while aberrantly localized proteins linked closely to human diseases [61, 62], such as Annexin A1 translocated to nucleus to promote pro-inflammatory cytokine expression [63]. Cx43 translocated from cell membrane to cytoplasm accompanied by cell retraction [64]. Here we reported that the distribution of TRPV1 was translocated to the cell membrane after OGD. Considering the amount of TRPV1 channels in the plasma membrane was closely related to its ion channel function [65], we derived that TRPV1 translocation to the cell membrane fully functions after HIBD, and what kind of factors mediated this translocation deserves discussion, as shown in Fig. 6. Besides, astrocytic TRPV1 could directly promote neuronal excitability after OGD, as found in Fig. 5, indicating it might be a crucial junction between astrocytes and neurons. TRPV1 increased excitatory neurotransmission by promoting proinflammatory responses in microglia [66, 67]. However, whether astrocytic TRPV1 is involved in the formation, release, or recognition of neurotransmitters thus affecting neuronal excitability deserves further discussion.

## Conclusions

In summary, TRPV1 activation directly affects the function of astrocytes after HI, promoting astrocyte migration to facilitate the pro-inflammatory factors dispersion and infiltration into the vicinity of neurons thus promoting the neuron excitability and ultimately accelerating onset of seizures. These indicate that astrocytic TRPV1 is not only a switch of pro-inflammatory factor but also a susceptibility gene for the induction of epilepsy following HIBD. Our data revealed the role of TRPV1 channels in seizure generation after HIBD, which may be a promising way to develop an effective immune therapy, such as broadening anti-inflammatory drug screening and designing clinical strategies for HIBD-induced epilepsy.

## Supplementary information

**Additional file 1.** Supplementary figure

## Data Availability

All raw data used in this manuscript are available from the corresponding author on reasonable request.

## Abbreviations

[Ca2+]i: Intracellular Ca^2+^ concentration
ARG-1: Arginase-1
DMEM: Dulbecco’s modified Eagle’s medium
EEG: Electroencephalography
GFAP: Glial fibrillary acidic protein
HBSS: Hank’s balanced salt solution
HIBD: Hypoxia-ischemia brain damage
IF: Immunofluorescence
IL-1β: Interleukin 1β
IL-6: Interleukin 6
iNOS: Inducible nitric oxide synthase
OGD: Oxygen-glucose deprivation
P0: Postnatal day zero
PBS: Phosphate-buffered saline
PCC: Pearson correlation coefficient
PTZ: Pentylenetetrazole
TNF: Tumor necrosis factor
TRPV1: Transient receptor potential vanilloid
WT: Wild type
