# Supplementary figures and images for: TRPV1 translocated to astrocytic membrane to promote migration and inflammatory infiltration thus promotes epilepsy after hypoxic ischemia in immature brain
Source: J Neuroinflammation. 2019 Nov 13;16:214. doi: 10.1186/s12974-019-1618-x (PMC6852893; doi:10.1186/s12974-019-1618-x)

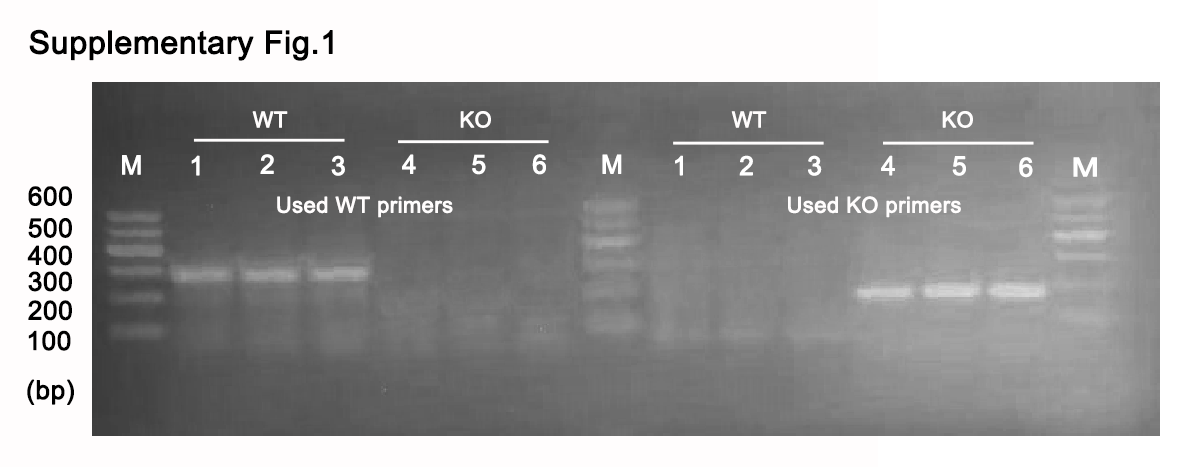

Supplement: Supplementary file 1 — Additional file 1. Supplementary figure [file 12974_2019_1618_MOESM1_ESM.tif]
